# Factors of parental investment in the home language environment in peri-urban China: A mixed methods study

**DOI:** 10.1371/journal.pone.0294158

**Published:** 2023-11-13

**Authors:** Tianli Feng, Jingruo Guo, Sarah-Eve Dill, Dongming Zhang, Yuchen Liu, Yue Ma, Lucy Pappas, Scott Rozelle

**Affiliations:** 1 School of Management and Economics, University of Electronic Science and Technology of China, Chengdu, Sichuan, China; 2 Stanford Center on China’s Economy and Institutions, Freeman Spogli Institute for International Studies, Stanford University, Palo Alto, California, United States of America; Tallinn University: Tallinna Ulikool, ESTONIA

## Abstract

The home language environment is a critical point of investment in early language skills. However, few studies have quantitatively measured the home language environment of low-socioeconomic-status households in non-western settings. This mixed methods study describes the home language environment and early child language skills among households in a low-socioeconomic-status, peri-urban district of Chengdu, China, and identifies factors influencing parental investment in the home language environment. Audio recordings were collected from 81 peri-urban households with children ages 18–24 months and analysed using the Language Environment Analysis (LENA^TM^) system. The Mandarin version of the MacArthur-Bates Communicative Development Inventory was administered to each child’s primary caregiver. The quantitative results revealed large variation in home language environments and child language skills among the sample, with relatively low average scores when compared to other Chinese samples. Qualitative interviews with a subset of 31 caregivers revealed that many caregivers face constraints on their knowledge of interactive parenting, compounded, in some households, by time constraints due to work or household responsibilities. The findings indicate a need for increased sources of credible parenting information for peri-urban caregivers of young children to promote investment in the home language environment.

## Introduction

Parental investment in the early home language environment is a key input in the development of language skills, which set the foundation for lifelong outcomes. The majority of brain development occurs within the first three years of life, making it a critical window for language development [1,2]. This is supported by multiple studies in Western populations, which have shown that children whose parents invest more in the home language environment, through increased rates of linguistic input and interactions, during this critical period tend to learn vocabulary at faster rates, show increased processing speeds, and develop stronger overall language and cognitive skills [3–6]. In turn, stronger early language skills lead to increased school readiness, stronger academic achievement, and higher academic attainment in the long-term [7–9].

Recent research on early language development has noted the diversity of linguistic and cultural contexts that influence language learning environments around the world, including patterns of everyday life, household organization, and community structures [10–13]. Differences in values, beliefs, and practices all influence early language and learning [10]. However, the literature has also identified disparities in investments between high and low socioeconomic status (SES) groups, which can lead to persistent disparities in later life. The literature has found several cases where children from lower SES households hear fewer words from adults and engage in fewer conversations with adults than their higher SES peers [3,5,6,14–18]. Evidence also suggests that children from lower SES backgrounds may be at risk of delayed development in their language skills when compared to children from high-SES backgrounds [3,19], due to higher exposure to risk factors prevalent in lower SES settings, such as infectious disease, malnutrition, poverty, and low availability of high-quality healthcare and educational resources [20].

Concerns about under-investment in the early home language environment are particularly acute in low- and middle-income country (LMIC) settings, where rates of developmental delay may be higher than expected in a healthy population. One study of nearly 100,000 children in 35 LMICs found evidence that approximately one-third of all 3- and 4-year-old children in LMICs were failing to meet basic cognitive milestones in 2010 [21], and a 2017 study found that approximately 43% of children under the age of five in LMICs are at risk of early developmental delays [22]. While rapid economic development may contribute to reducing these delays, developmental delays may persist in areas of the world facing continued high exposure to risk factors such as infectious disease, malnutrition, poverty, and low availability of high-quality healthcare and educational resources, which have previously been shown to have higher concentrations of low development scores [21]. However, to date the majority of research on language development among low-SES groups has been concentrated in high-income Western countries, and comparatively less is known about the home language environment among low-SES households in low- and middle-income countries (LMICs), where rates of developmental delay among young children tend to be significantly higher [21].

China is a middle-income country that has experienced rapid economic growth in the past four decades [23]; however, significant disparities remain between urban households (which typically have higher SES) and households from lower income, less developed rural environments [24–26]. One key disparity is in early childhood development (ECD): whereas children under age three in urban areas have rates of developmental delays around 15% [27], a systematic review of child development before age three among rural populations across China found rates of cognitive delay (defined as one standard deviation below the mean of a healthy population) around 45% and rates of language delay around 46% [28]. Studies examining the source of those delays have found that a large fraction of rural caregivers do not regularly engage in interactive parent-child activities such as reading, telling stories and singing [29], which are known to stimulate early cognitive and language development [30–34].

There is also evidence of rural-urban disparities in the home language environment: a series of studies describing and comparing the home language environments of young children in low-SES households in rural Shaanxi Province to that of high-SES households in urban Shanghai demonstrated that rural children overheard significantly less adult speech and have fewer adult-child verbal interactions compared to their urban peers [35–37], demonstrated that rural children overhear significantly less speech and have fewer adult-child verbal interactions compared to their urban peers. Another study among low SES rural households in two southwestern counties of China similarly found lower counts of adult words and adult-child conversation [38]. The same study additionally found that adult-child conversation significantly predicted child language skill, even after controlling for other measures of parental stimulation [38]. This was supported by another study which also found measures of the home language environment to be significantly correlated with child language skills after controlling for other indicators of parental stimulation [39].

In addition to differences between urban and rural areas of China, a small amount of research has identified differences within China’s cities between established urban households and rural households living on the urban periphery. In China’s rapid urban expansion, peri-urban spaces known as “villages-within-cities” (*chengzhongcun*) have emerged around urban centres as communities for households from rural backgrounds [40,41]. Residents of these communities typically fall into one of two groups: newly urbanized farmers and rural migrants [42,43]. Urbanized farmers are formerly-rural local residents whose farmland has been lost to urbanization [44]. Rural migrants are households that have out-migrated from their rural hometowns to urban centres for work, a group that now accounts more than 200 million people in China [40,45]. Although peri-urban families typically have higher levels of income and education than those in rural areas [46,47], both rural and peri-urban households have lower SES (in terms of income and education) than their established urban counterparts [26,48,49]. Peri-urban communities can therefore be considered low SES, quasi-rural populations within recently urbanized settings [40,41].

Although peri-urban households show improved socioeconomic outcomes compared to rural households in China, preliminary evidence suggests that peri-urban children face similar disparities in language skills and the home language environment. One study found that 39% of rural migrant children living in urban China exhibit language delays [29], which is more than double the 15% rate in a healthy population [50]. The study also found that in the day before the survey, 52% of caregivers had sang songs with their child, 29% had told a story to their child, and only 21% had read a book with their child [29]. Two recent studies of the home language environment based on a sample of rural migrant and urbanized farmer households in peri-urban China found even lower counts of adult-child verbal interaction than previously observed in poor rural areas, with the home language environment significantly predicting child vocabulary [51,52].

From an economic perspective, high rates of under-investment in key language skills among China’s young children raises concerns for economic growth and social well-being. Sustained economic growth relies on a skilled workforce that can maintain competitiveness in the global market [20,21]. Countries like China, which aim to transition from a middle-income country to a high-income country, must have a large share of the workforce who have sufficient skills to support the transition to an economy based primarily on high-skill, high-income industry [53–56]. However, research indicates that adults raised in disadvantaged contexts are less likely to attain the necessary skills to enter high-skill workforces [5,14]. In other words, there is a “normative” set of skills that is highly valued for economies in transition [57–59]. Moreover, these “normative” skills are developed through having sufficiently high levels of cognition and language which are established in the first years of life [57,58]. Thus, identifying targets to positively influence ECD may therefore improve widespread human capital and sustained economic growth over time.

Several possible factors that may influence investments in the home language environment have been identified in the literature to date, including economic constraints, time constraints and knowledge constraints. Studies both internationally and in China have pointed to poverty as a potential factor that influences how caregivers invest in their children, with poverty creating a financial stress or inability on caregivers to provide stimulating books and toys to their children [60–62]. Another possible factor of parental investment is constraints on caregiver time due to work or responsibilities inside the home. Time constraints may be particularly salient for peri-urban households due to multiple family members working [25,63–65]. Finally, knowledge of stimulating parenting has also been identified as a significant factor of parental investments in the home language environment. Studies in LMICs, including China, have found that the knowledge on ECD and stimulating parenting practices that many low-SES caregivers have access to does not accurately reflect findings on what parenting practices positively contribute to developmental outcomes [28,66–70]. It is highly likely that these constraints are interrelated or interdependent: for example, caregivers in poor households may face time constraints due to their poor economic condition.

Recent research has begun to identify factors associated with the home language environment among low-SES peri-urban households. Feng et al. (2023) found that children experienced more adult speech and verbal interactions when they are older, their mothers are employed, they are female, and when their household has a higher level of assets [51]. These results suggest that economic constraints may limit the home language environment. Another study drawing on the same sample examined the role of parental self-efficacy in child cognitive and language skills, finding that although self-efficacy was positively correlated with child cognitive skills, there was no significant correlation with language skill [52]. One limitation of these studies, however, is their use of cross-sectional survey data. Although these data allow researchers to quantitatively identify predictors of the home language environment, they are unable to capture the challenges to caregiver investment in the home language environment in their day-to-day lives. Understanding how different constraints shape the daily investments of caregivers can be better accomplished through qualitative methods such as interviews, which allow caregivers to describe how they think about and enact their parental investments.

To fill this gap in the literature, we conduct a mixed methods study to understand the home language environment and early language skill of young children aged 18–24 months in peri-urban China and identify factors that shape caregiver investment in the home language environment. We employ a mixed methods approach to fulfil three objectives. First, we describe the home language environment of peri-urban households using quantitative measures of the home language environment generated through Language Environment Analysis [71]. Second, we quantitatively describe the language skill (vocabulary) of young children in peri-urban households. Finally, through qualitative interviews with a subset of caregivers in the quantitative sample, we examine three hypothesized factors that may influence parental investment in the home language environment (economic constraints, time constraints and knowledge constraints).

## Theoretical framework

**Fig 1:**
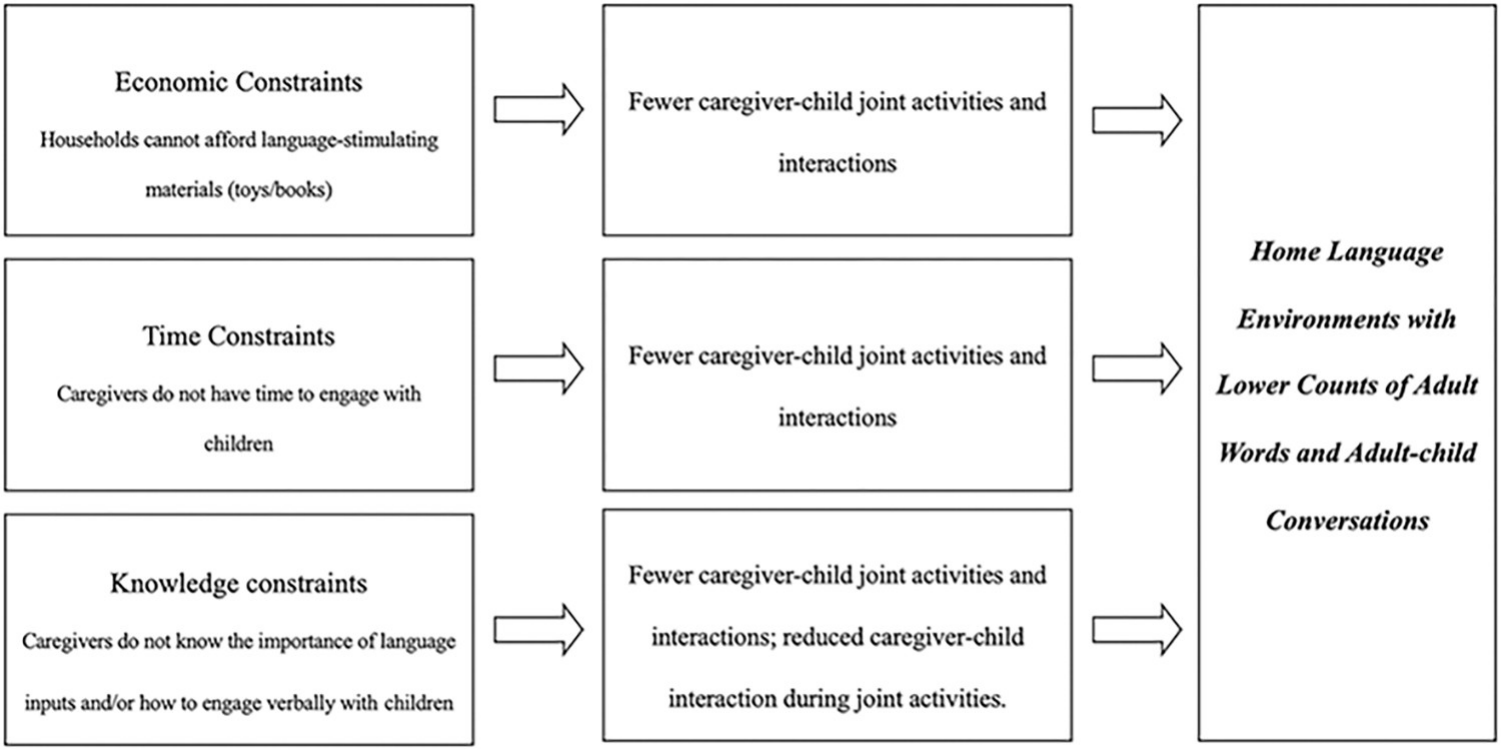
Model of factors of parental investment in the home language environment.

The literature internationally and in China has identified some significant correlates of parental engagement. These factors may similarly apply to parental investment in the home language environment. In the qualitative portion of this study, we examine three hypothesized environmental and caregiver factors that may influence investments in the home language environment among households in peri-urban China: economic constraints, time constraints and knowledge constraints (Fig 1).

### Hypothesis 1: Economic constraints

The first potential factor of caregiver investment in the home language environment is economic constraints on peri-urban households. The international literature has found that caregivers in lower SES households tend to have lower levels of parental engagement and poorer ECD outcomes than higher SES families [19,60,72]. In the literature from Western, high-income countries, research has also documented differences in linguistic input between lower and higher SES households, with several studies suggesting that lower SES caregivers respond less frequently to their children and use less varied vocabulary, syntax and gestures [3,6,14,15,62,73]. In at least a subset of these papers, the literature has suggested that such differences may be due to economic constraints that prevent caregivers from purchasing toys, books and other materials that help to foster parent-child interactions [60,62], and some studies suggest that financial stress may also limit the capacity of caregivers to engage with their children [61].

In China, similar to the international literature, studies have found large differences in parental investments and ECD outcomes between high-SES urban families and low-SES rural families [29,74–77]. There is also preliminary evidence of large differences between high-SES urban households and low-SES rural households in terms of the home language environment [35,36]. There is reason to believe the same is true for peri-urban households, as studies of rural migrants in urban China have found similar ECD outcomes to rural communities [29]. Moreover, although peri-urban communities have higher SES than rural areas, they may face a higher cost of living as well and more visible socioeconomic inequalities between established urban households and peri-urban households from rural backgrounds [26,35,42,48,49,78,79]. For this reason, our first hypothesis is that constraints on economic resources limit caregivers in their abilities to invest in the home language environment.

### Hypothesis 2: Time constraints

Our second hypothesized factor of parental investment in the home language environment is the constraint on the time caregivers have to spend with their children. The literature, both internationally and in China, has established that time investments in stimulating parent-child interactions are essential to healthy ECD [28,80,81]. Similarly, a linguistic input in the home language environment is predicated on caregivers investing time into speaking with their children. Time constraints have been frequently cited as a challenge for low-SES caregivers, as households may need to prioritize time spent working or caring for other dimensions of the overall household over caregiver-child interaction time [82,83].

In peri-urban China, there are two possible sources of time constraints for caregivers of young children: employment outside of the home and responsibilities within the home. First, employment outside of the home may limit the time caregivers (and other adults at home) have available to engage with children. This may be particularly true for rural migrants, who typically work longer hours than their urban counterparts [25,64,65]. Additionally, due to the higher costs of living in urban areas compared to rural areas, peri-urban households typically have more family members working outside of the home than rural households [84,85], meaning that there are fewer adults at home to engage verbally with the child.

The second possible source of time constraints comes from other household responsibilities (beyond caregiving). Studies from rural areas of China have found that the primary caregivers of young children (typically mothers or grandmothers) are most often responsible for household chores, caring for sick or elderly family members, and caring for older children [66,86]. The same demands may also be at play in peri-urban households. Moreover, in households with multiple family members working, the child’s primary caregiver may be both the only person managing the household and the only adult available to interact with the child. For these reasons, we hypothesize that caregivers may be unable to invest large amounts of time in contributing to the home language environment because they do not have enough time to consistently speak or interact with their children.

### Hypothesis 3: Knowledge constraints

The final potential factor of parental investment in the home language environment is constraints on the knowledge of caregivers regarding child development and parenting. The theoretical and empirical literature on parenting has shown that knowledge and beliefs about child development broadly inform parenting practices and the ways caregivers interpret and respond to caregiving situations [87–90]. Caregivers tend to speak and interact more frequently with their children when they believe parent-child interactions to be important for their children’s development [91–93]. However, several studies have found that caregivers of young children tend to underestimate the importance of parent-child interactions in the earliest years [94,95]. In LMICs, a number of studies have pointed to inaccurate parenting knowledge as one of the major drivers of poor ECD [96,97].

A number of studies on parenting in rural China have pointed to poor knowledge of parenting among rural caregivers. A recent mixed methods study of caregiver engagement in reading with young children in rural China found that the majority of caregivers do not read with their children because they lack actionable knowledge about effective early childhood reading practices [66]. A systematic review of ECD interventions in rural China also found evidence that interventions improved parental investments and parent-child interactions by influencing the knowledge and beliefs of caregivers [28]. These findings also are supported by evidence that many rural caregivers do not have access to high-quality parenting information sources: in a study of parenting practices in rural China, Luo, Jia (74) found that only 15% of caregivers relied on formal information sources such as local doctors or public health practitioners, and instead most commonly sought advice from family members and friends, particularly older generations whose parenting strategies may no longer be reliable (or applicable) in a rapidly developing society [74]. Given the rural backgrounds of peri-urban households in China, our third hypothesis is that caregivers investments in the home language environment are affected by incomplete or inaccurate knowledge about ECD and parenting.

Although we have described these three hypothesized factors separately, there is a high likelihood that economic, time and knowledge constraints are interrelated or interdependent. Caregivers in economically constrained households may face time constraints because they must work longer hours to make ends meet. Caregivers facing knowledge constraints may also experience economic constraints because they do not have education levels sufficient to access well-paying jobs. It is also possible that some factors may be more prevalent or influential among the sample. For example, a study of early childhood reading in rural China found that economic concerns were less salient as a constraint to interactive caregiver-child reading compared to knowledge constraints [66]. Understanding which factors play the most prominent role in influencing caregiver investments in the early home language environment can inform community interventions that leverage salient beliefs, values and practices to support healthy early childhood development.

## Materials and methods

The data used in both the quantitative and qualitative study were collected from a peri-urban district in eastern Chengdu, the capital city of Sichuan Province, China. Originally rural farmland, this district has experienced rapid urbanization in recent years as a part of Chengdu’s eastern expansion plan [98] and plan to develop a two-city economic circle with Chongqing [99]. As a result, the vast majority of local residents no longer own farmland, instead living as newly-urbanized residents. In addition to these urbanized farmers, of the 714,000 residents in the district, nearly one third (200,200) are rural migrants who have relocated from their rural hometowns for work [100]. The average per capita income of rural residents in the district is 27,742 RMB ($4,278), considerably lower than the 42,128 RMB ($6,496) average per capita income of urban residents in the district [100]. In this way, the sample region is relatively representative of China’s many peri-urban “villages-within-cities” in which low SES households from rural backgrounds now live and raise their children [40,41].

### Ethics statement

This study received ethical approval from the Stanford University Institutional Review Board (IRB Protocol 57196). Trained members of the field survey team obtained informed oral consent from all caregivers for their own and their children’s participation in the study, after introducing the research following a script in Mandarin Chinese and allowing for caregivers to ask questions. All participants understood that their home language environment data would be recorded and analyzed for the purposes of this study.

### Quantitative methods

#### Sampling strategy

Sample households were selected using a two-step protocol. First, the research team obtained a list of children between 18–24 months from two local hospitals in the district. We chose this age range because it is one of the most crucial developmental periods for language learning in young children. Whereas most 12-month-old infants are just beginning to speak and acquire new words at a slow pace [101], many toddlers show a “vocabulary burst” around 18 months of age, shifting to a much faster rate of language acquisition [102]. By 18 months, many toddlers can also distinguish patterns of grammar in familiar passages [103], and by 24 months, toddlers can typically produce 200 to 500 words [104]. Additionally, because of the rapid pace of language acquisition for many children in this window, research has also found relatively large variation in language processing skills and expressive speech among 18- to 24-month-old children [62,105]. This age range therefore represents a period of particular interest when examining variations in parental investment in the home language environment and potential sources of variation.

Second, members of the research team randomly screened households from the list via phone calls to ensure they met the criteria to be considered urbanized farmers or rural migrants according to the Chinese government, and all eligible households were invited to enroll in the study. Households were considered urbanized farmers if a.) the legal residence of either parent had to be within the sample district; b.) all or part of the household’s farmland must have been acquired by the government to be used for urban development; and c.) any remaining land owned by the household must average to less than 200 square meters per adult family member [44]. Household were considered rural migrants if a.) the legal residence of either parent in the household was from a rural area outside of Chengdu Municipality; and b.) at least one parent had lived in the sample district for 6 months or more [106]. Following this protocol, 109 eligible households were enrolled in the study, and 81 households allowed the research team to collect recordings of the home language environment. Some households were unwilling to follow the LENA recording protocol, and some households had social events planned that did not allow them to record their typical days. A dependent t-test was used to compare the characteristics of participants in the LENA consent and reject sub-samples (S1 Table). No significant differences in any control variables were found between the two groups. The final quantitative sample therefore consists of 81 households with children between 18–24 months in a peri-urban district in Sichuan.

#### Data collection

Data were collected in July 2020 by trained enumerators following a 4-day data collection protocol. On the first day, enumerators conducted structured survey interviews with each child’s primary caregiver at their home or at the local hospital. Following the survey, caregivers were instructed on how to use the LENA recording devices. Families used the LENA devices to record the child’s home language environment on the second and third days. On the fourth day, the research team retrieved the LENA devices and conducted pick-up interviews to ensure compliance with the data collection protocol. Following this protocol, the research team was able to collect data on the home language environment, child language skill, and child and household characteristics.

We assessed each child’s home language environment using the Language Environment Analysis [71] system [107,108]. LENA is a small digital recorder that records everything a child hears or utters for up to 16 hours. LENA has been shown to be valid and reliable in Mandarin [35,109], as well as a number of other languages [110–113]. In this study, each participating household was given a LENA recording device, which was placed in the chest pocket of a specialized LENA shirt that the child wore throughout the day. Caregivers were instructed to record two 16-hour days that represented their child’s typical routine, only removing the recorder when the child bathed or slept, and to charge the recorder in between the first and second days of recording. The average LENA recording durations is 1,766.64 minutes (about 29.44 hours), with a standard error of 138.2405.

The two-day recordings were standardized into 12-hour data before being analysed using LENA software [114], and the reported values are averaged across both days. To adjust for skewing commonly seen with count data, we followed Ma et al. [36] in using Chebyshev polynomials transformation to normalize the distribution of all LENA-generated measures [115]. We chose the final Chebyshev polynomials model used to transform the data by using least absolute shrinkage and selection operator (LASSO) regression models [116]. We then predicted residuals using the final Chebyshev polynomials model. Finally, we estimated residualized count variables from the transformed data and rescaled residualized count variables back to the original count metric.

LENA generates three primary measures: *adult word count* (AWC), the number of words spoken to or near the child by an adult; *conversational turn count* (CTC), the number of adult-child alternations in conversation; and *child vocalization vount* (CVC), the number of words or word-like utterances made by the index child. In this study, AWC and CTC are used as measures of the home language environment. CVC is used as one of two measures of child langauge skill. CVC has been used as a measure of child language skill in the literature [15], as child vocalizations are highly predictive of future language skills [117,118].

The second measure used to the assess child language skill is the short-form Mandarin version of MacArthur-Bates Communicative Development Inventory (CDI) for 16 to 30-month-old children. This version of the CDI is designed to measure expressive language [99], and it has been validated in Mandarin-speaking populations among children in our sample age range of 18–24 months [119]. It is important to note that the CDI measures child language skill specifically in the domain of vocabulary. The CDI contains an inventory of 113 words; during the survey interviews, enumerators asked caregivers whether their child could speak each word in the inventory. It can be difficult for caregivers to judge, based on whether their child can speak a word, whether their child understands the meaning of a word [119]. Following recommendations from the user’s guide for the Mandarin version of the CDI [119], when a caregiver answered affirmatively that their child could speak a word, enumerators were trained to ask the caregiver to provide an example of the child using the word to determine whether the child understood the word.

Finally, we collected information on the demographic characteristics of sample children and households. Child characteristics include the child’s age in months, gender, and whether the child was born prematurely. Household characteristics include whether the mother was the child’s primary caregiver, maternal age, whether the child’s mother has a job, maternal and paternal education levels, whether the child’s father lived at home during most of the previous year, and the value of household assets. To measure household assets, we created a household asset index using polychronic principal components analysis based on whether each household owned or had access to running water, a toilet, a water heater, a washing machine, a computer, Internet, a refrigerator, air conditioning, a motorcycle or electric bicycle, and a car or truck.

#### Statistical analysis

We perform descriptive analysis for all quantitative measures. First, we present the summary statistics for child and household demographic characteristics. We then report the means and standard deviations [120] for the home language environment (AWC and CTC) and child language skill (CVC and CDI). We report means and SD for the full sample, as well as separately for the top, middle and bottom terciles of the distribution for each measure.

### Qualitative methods

In addition to the quantitative data, we also gathered qualitative data to better understand the factors that influence caregivers’ investments in the home language environment of their young children. To do so, a subset of children and caregivers from the quantitative sample were invited to participate in semi-structured qualitative interviews. In total, we interviewed 31 primary caregivers across the distribution of the home language environment and child language skills. An independent-sample t-test of the qualitative subsample and the full quantitative sample. The results, (S2 Table) found no significant differences between the qualitative subsample and the full quantitative sample in terms of key demographic and socioeconomic characteristics.

The qualitative interviews were conducted in each participant’s home by trained qualitative interviewers. Interviewers asked a set of open-ended questions about caregivers’ childrearing experiences; knowledge, attitudes and beliefs about child development and parenting; the economic conditions of the household; the responsibilities and time demands on caregivers and other family members; and family dynamics within the household. While the interview protocol served as the primary guide, interviewers were free to ask follow-up questions based on the answers of the respondents.

In addition to the qualitative interviews, we also collected qualitative observational data on the home environment and caregiver-child interactions during the semi-structured interviews. Members of the research team recorded observations of each family’s home, material conditions, and children’s books and toys to better understand the economic conditions of households. In addition, to understand how caregivers engage with their children, interviewers also asked caregivers to demonstrate how they interact with their children (e.g., reading or playing together) and recorded their observations of parenting practices and parent-child interactions during the demonstrations.

The qualitative (interview and observational) data were analysed following a matrix coding procedure. First, data for each respondent were divided into three dimensions in accordance with our three hypothesized factors identified in the literature. Each factor was then coded as 0 or 1, with 1 indicating that the factor had a negative impact on the home language environment of the sample household and 0 indicating no negative impact. Finally, we counted the number of respondents who had received a code of 1 for each hypothesized factor to identify which factors predominantly contribute to lower counts of AWC, CTC, and CVC in the home language environments among peri-urban households.

## Results

### Quantitative findings

Table 1 presents the basic demographic characteristics of children and households in our sample. At the time of the survey, the average age of sample children was roughly 21 months. About 57% of children were male, and 10% were premature. In 57% of sample households, the mother was the child’s the primary caregiver; among the remaining 43% of households, the child’s maternal or paternal grandmother was typically the primary caregiver. The average age of mothers in the sample was about 29 years old. Approximately 77% of both mothers and fathers had completed high school, and about 63% of mothers were employed at the time of the survey. There was an average of three adults living in each sample household, and about 79% of fathers lived at home for the majority of the previous year.

**Table 1:** Descriptive statistics of child and household characteristics (N = 81).

	Mean / n	SD / %
	(1)^a^	(2)^a^
***Child characteristics***		
Age (months)		
18–20 months	37	46%
1–22 months	32	39%
23–24 months	12	15%
Male infant	46	57%
Premature birth	8	10%
***Household characteristics***		
Mother is primary caregiver	46	57%
Maternal age (years)	29.11	4.69
Maternal has ≥9 years of education	62	77%
Father has ≥9 years of education	64	79%
Mother has a job	51	63%
Father lived at home most of last year	64	79%
Number of adults in household	3.05	1.01
Number of siblings in household	0.12	0.33
Household asset index (score)^b^	0.00	1.28
Urbanized farmer household	47	58%

^a^ Data are presented in mean and standard deviation [120] for continuous variables and in frequency (n) and percentage for binary variables.

^b^ The household asset index was generated using principal components analysis based on whether the household owned or had access to running water, a flush toilet, a water heater, a washing machine, a computer, Internet, a refrigerator, air conditioning, a motorcycle and a car/truck.

Table 2 presents the measures of the home language environment (AWC and CTC) and child language skill (CDI) among the sample. The mean [120] for AWC and CTC scores among the sample are 12,354 (5,522) and 482 (262). The mean [120] for CVC is 1,734 (749), and the mean [120] for CDI is 37 (27). The large standard deviations in the home language environment results also indicate a large degree of variation in both the home language environment and child language skill across households. This is supported by large differences among the top, middle and bottom terciles of the sample. The mean AWC and CTC for the top third of households in our sample (18,532 and 777, respectively) were both nearly three times higher than those of the bottom third (6,986 and 247, respectively). Similarly, the average CDI score for the top third of the sample (72.5) is six times greater than the mean score of the bottom third (12.5). A supplemental analysis of correlations between AWC, CTC and CDI was conducted among the sample (S3 Table), showing that AWC and CTC are significantly correlated with child vocabulary (CDI). In other words, lower investment in the home lagnauge environment is associated with lower child vocabulary. Taken together, these results suggest that a large share of peri-urban households may not be providing sufficient language inputs to support their children’s language development. The next section of the paper seeks to answer why that is.

**Table 2:** Descriptive statistics of LENA outcomes.

	Full sample (N = 81)	Top 1/3 (n = 27)	Middle 1/3 (n = 27)	Bottom 1/3 (n = 27)
	(1)	(2)	[121]	(4)
***Home language environment***				
AWC^b^, mean [120]	12,354	18,532	11,561	6,968
	(5,522)	(4,270)	(1,652)	(1,564)
CTC^b^, mean [120]	482	777	422	247
	(262)	(226)	(76)	(52)
***Child language skill***				
CVC^b^, mean [120]	1,734	2,546	1,564	1,092
	(749)	(708)	(164)	(238)
CDI^b^, mean [120]	37	68	33	10
	(27)	(19)	(8)	(6)

^a^ Data are presented in mean and standard deviation [120].

^b^ “AWC” is defined as Adult Word Count, “CTC” is defined as Conversational Turn Count, CVC is defined as Child Vocalization Count and “CDI” is defined as Communicative Development Inventories.

### Qualitative findings

The quantitative findings reveal large variation in measures of the home language environment and child language skills among the sample. These findings suggest that although some households report high counts of AWC, CTC, CVC, and CDI, a large share of peri-urban households’ home language environment and child language skill scores fall below the average. These findings lead us to ask: What factors may be preventing caregivers (and other family members) from engaging in higher rates of language interactions with their children? To answer this question, we draw on qualitative interviews with a subset of caregivers to examine three factors that we hypothesize to influence how caregivers invest in the home language environment: economic constraints, time constraints and knowledge constraints.

### Hypothesis 1: Economic constraints

Our first hypothesis posits that caregiver investments in the home language environment are limited due to financial limitations. Counter to this hypothesis, however, our qualitative interviews reveal that in general, caregivers can comfortably afford to invest in their children. Most respondents reported that they could buy whatever they wanted for their children without having to tighten their belts.

“He [my grandson] started reading last year, now we have books sent to our home every month. Books ordered for him [to read]. They [my son and daughter-in-law] ordered a batch of books that are suitable for him to read at different ages. A box of books is sent to him every month.” (Grandmother-11144)

“We buy what she wants. We never say no. We would buy whatever she wants, like toys, so long as she likes. Whatever her sister wants, we would buy for her too.” (Grandmother-11116)

“We will buy any book that looks good. When her mother sees good books, she will also buy them as long as she likes them. Her grandparents sometimes also buy her toys. Anyway, we all buy books for her.” (11106)

“Whatever he wants, his parents will buy for him. Mom and Dad would buy him whatever [he likes], like the toys.” (Grandfather-11041)

From these responses, financial constraints do not appear to limit caregivers from investing in stimulating materials for their young children. Supporting this, the observational data found that more than half of the surveyed families owned age-appropriate books, and nearly all families have more than ten toys. This leads us to believe that economic constraints are not the primary factor effecting caregiver investments in their home language environments.

### Hypothesis 2: Time constraints

Our second hypothesis is that time constraints, due to employment or household responsibilities, may influence how much caregivers can invest in the home language environment. In our qualitative interviews, nearly half of caregivers (14 of 31) reported having insufficient time to interact with their children, suggesting that constraints on caregiver time may be a factor of investments in the home language environment for at least some households. Several interviewees specifically mentioned constraints on the time of mothers due to employment outside of the home.

“The child’s mother started working when she was one year old. I usually take care of the child when the mother goes to work. She takes over when she comes back at night, but she doesn’t have much time because she comes back late … I take on the large part [of caregiving]. There are no other people [helping take care of the child].” (Grandmother-11109)

“[The baby’s] mother has only one day off each week, and the off-day is not fixed. Also, her mother usually leaves early and returns late. Sometimes when her mother leaves for work, [the baby] is still asleep, and by the time she comes back home, the baby is asleep again.” (Grandmother-11032)

“Our shop needs people to watch it as long as it is open, so it’s difficult to take him [my baby] out to play. Sometimes when I think about it, I feel guilty… [but] we are in the service industry, and it’s hard to tell when the customers will come … we cannot close the shop all day.” (Mother-11100)

In addition, with multiple family members (including mothers) working, some caregivers of young children at home reported feeling overwhelmed by housework and childcare, particularly in households with more than one child:

“I can do nothing about [interacting with my child] because I am the only adult in the house. Sometimes he will play with toys by himself, and sometimes he will follow me and go to the kitchen. There are only two of us [in the house].” (Grandmother-11109)

“I have to do the housework, so I don’t have much time to interact with the baby. I might be cooking or tidying things up, so I don’t have time to play with him.” (Mother-11120)

“My older son is sometimes disobedient at home, and he must be supervised in doing his homework. My mom … her energy is limited, and she has to cook for us, so I have to supervise my older son’s homework, so we just don’t have that much energy to look after the younger one or to talk to him.” (Mother-11079)

“I need to take care of two kids, so [I] cannot pay attention to both of them so carefully… But the elder kid will be sent to kindergarten in the second half of the year, and then I will have more time to interact with him [the younger child]. Now, I can only pay partial attention to him.” (Grandmother-11064)

These responses suggest that time constraints do present an influential factor of caregiver investment in the home language environment for many households. Demands on caregivers’ time, both due to employment outside the home and responsibilities within the home, distract caregivers from engaging in language-stimulating activities or parent-child interactions, despite having age-appropriate books and toys.

Nevertheless, although half of caregivers reported time constraints to limit their verbal interactions with children, the other half of caregivers stated that they do have enough time to interact with their children:

“The two of us were together all day. We [interact with each other] if there are no special things.” (Mother-11027)

“I am retired; I stopped working just because I need to take care of the baby. (Grandmother-11032)

“[Do I have] enough time? My time should be sufficient… I just take care of my kid at home and accompany him.” (Mother-11002)

The statements above suggest that time constraints may be a factor of lower rates of investment in some households, but not all. In households with multiple family members working, time constraints affect parental investments and caregiver-child interactions. For households where the caregiver is only responsible for childcare, however, time constraints do not present affect the investments in caregiver-child interactions or the home language environment.

### Hypothesis 3: Knowledge constraints

Our third hypothesis suggests that caregivers’ investments in the home language environment are linked to the knowledge they have about early language development, the importance of the home language environment, and how to engage in verbal interactions with their children. Specifically, we defined caregivers with less accurate/complete knowledge whenever one of his answers to our questions is incorrect. Our qualitative interviews find strong evidence that misconceptions about parenting and child development influenced the investments made by caregivers. Although all caregivers stated that they believed the home language environment and parent-child interactions to be important to their children’s development, a large share of caregivers doubted the value of language inputs from adults during their child’s earliest years. When asked to name the most important aspects of a child’s development before age three, more than half of caregivers (16 of 31) did not mention language development at all, and a similar share (14 of 31) instead listed physical health as the most valuable investment during early childhood.

“I think my child is still young, so physical health is more important [than other things].” (Mother-11034)

“It must be the physical health [that is most important]. Physical health is definitely a must, because we cannot do those other things well unless we are healthy, and then there is no more.” (Mother-11042)

“The important thing must be good health … Everything is ok as along as the baby doesn’t get sick.” (Grandfather-11046)

“[My grandson] must be eating well and drinking well first. He must eat well, then he can be [healthy].” (Grandmother-11027)

“Physical health must be the first priority regardless of time.” (Mother-11023)

Moreover, when caregivers were specifically asked about language development in the first three years, many stated they believed their child to be too young to learn or benefit from verbal interactions with adults:

“For example, my friend would read aloud to her daughter when she sees a sign, something like ‘picking flowers and plants is not allowed.’ I wondered at the time, since [her daughter] cannot understand what she was saying, why explain so much to her?” (Mother-11120)

The same mother continued later in the interview:

“[My friends] started such input [talking to children] at an early age. I thought that [my child] was still young, and he couldn’t understand it, so I didn’t start to enlighten him.” (Mother-11120)

Other mothers and grandmother-caregivers expressed similar feelings:

“In terms of language, he doesn’t understand it anyway.” (Mother-11100)

“Because he [my son] can’t speak yet, I feel like there are a lot of words he might not understand.” (Mother-11077)

“I don’t think she can understand what I say to her, because she is too young.” (Grandmother-11116)

Finally, a number of caregivers also believed that interactions with other children would be more beneficial to their child’s development than language inputs from adults.

“I used to think my child may learn something in conversations with adults, but that doesn’t happen … I should take him out more to meet other children, let him talk more.” (Mother-11149)

“Contacting more with [other children] will definitely help her develop her language ability. [I] often take her out to get in touch with other children.” (Grandmother-11019)

“Babies aged 1–3 years are in the imitating period and imitate others a lot, so I said I want to enroll him in kindergarten. [In kindergarten], different babies have different skills that they can share with each other. I will do that when he is three years old.” (Mother-11100)

“I think that he has a strong imitation ability when he plays with [other] kids, because he learned how to say ‘brother and sister’ from his buddies. So, I think playing with other kids is good for him to learn things.” (Mother-11120)

Such statements suggest that despite stating positive beliefs about parent-child interactions, many parents undervalue the importance of their own investments in the home language environment and interactions with their children in the first three years of life.

In addition to misconceptions about the importance of parental-child interactions for ECD, our interviews revealed that many caregivers do not know how to interact with their children in a stimulating way. When asked questions such as “How do you interact with your child?”, only 9 of 31 caregivers demonstrated knowledge of interactive parenting practices, and many caregivers instead described passively observing their child rather than directly interacting with them.

“If he wants to play something, I go with him. When he plays, I watch him play, or just follow him [to ensure his safety] … He’s older now, and he can play by himself, so I let him play by himself” (Mother-11120)

“Sometimes if she’d like to play in the living room, I let her stay in the living room; if [she] would like to be in kitchen, I let her stay in the kitchen. Sometimes, when I’m cooking, she will open the cabinet [in the kitchen] by herself and take some things out to play by herself.” (Mother-11106)

“Sometimes I would cook, and I would interact with him when I’m free, or play with my cell phone. But basically [I just] keep an eye on him. I keep an eye on him all the time.” (Mother-11077)

“She [my baby’s grandmother] just takes the baby out to play together with other old women and children in the community. [The baby] plays together with a few friends similar to her age.” (Mother-11047)

“When [my child] gets up in the morning, I say good morning to her, and spend time with her … Sometimes she would bring books over and read it for a while by herself. Now she gets older, and when she gets up in the morning, she would run out and play outside by herself.” (Mother-11125)

These responses indicate that even when caregivers understand the importance of language interactions for language development and have the opportunity to engage in such interactions with their children, many caregivers do not know how to engage in stimulating parent-child interactions that support their children’s development. Taken together, knowledge constraints appear to be a significant factor of parental investment in the home language environment among peri-urban households.

What are the causes of knowledge constraints among caregivers? Our qualitative interviews reveal that many caregivers do not have reliable sources of parenting information. Less than a third of caregivers (7 out of 31) obtained their information from credible sources such as doctors, books or early childhood education centers, and nearly two thirds of caregivers (19 out 31) reported they received information from websites, social media, friends and family, or their own personal experience.

“I have no (specific information sources). Usually, when I encounter problems in my own parenting process, I look for answers on the Internet or [think about] the advice given by others. And if I have no problems with myself, I won’t look for the answer.” (Mother-11003)

“I ask my colleagues [for parenting advice], because their children are a little older than my child, and [they] tell me about what they have experienced.” (Mother-11047)

“Most of my information comes from my parents [the child’s grandparents].” (Father-11002)

“I read some [parenting articles] on the phone, and sometimes when going out, I would talk [about how to raise a child] with my friends, and sometimes my daughter also talks to me about it.” (Grandmother-11109)

The qualitative interviews also revealed differences between parent versus grandparent caregivers in terms of both parenting knowledge and sources of parenting information. Only 1 of 15 grandparent caregivers demonstrated accurate knowledge of child development and stimulating parenting, whereas 8 of 16 parents did so. Grandparents were also more likely to rely on their own intuition or experience to guide their parenting practices:

“I don’t get information online. It’s all my own experience. I have been a mother before, and now I’m a grandparent, so I’ve accumulated a lot of experience… [Taking care of a baby] should combine the old ways with the scientific ones. Both are important.” (Grandmother-11082)

“Generally, I get [parenting information] from others. My education level is relatively low, and so are my communication skills, so I only have a general sense [of what to do].” (Grandfather-11041)

“I raise my kid mainly based on my previous [parenting] experience.” (Grandmother-11116)

“We do not check online. His mother lets us teach him the way we want, and she believes that we would not educate the kid in a bad direction.” (Grandfather-11144)

This difference implies that the identity of a child’s primary caregivers could be an important factor in investment in the home language environment among our sample, as nearly 40% of primary caregivers in the quantitative sample are grandparents.

Taken together, our qualitative interviews point to knowledge constraints as the primary factor for most of the sample families’ investments in the home language environment. A large share of caregivers underestimates their role in early language interactions and language development, and many do not understand how to engage in language interactions with their children, due in large part to a lack of reliable information sources on parenting and ECD. In some households, this is also compounded by constraints on caregiver time: with multiple family members working, the burden of both household responsibilities and childrearing fall upon the primary caregiver, limiting their time to engage in language interactions with the child.

## Discussion

The goal of this mixed methods study was to understand the home language environment, early language skill and factors of parental investment in the home language environment among households from rural backgrounds living in peri-urban Chengdu, China. Drawing on quantitative survey and LENA data from 81 households, we described the home language environment and child language skill outcomes of sample children. Consistent with literature that households in peri-urban areas have higher SES than rural families [46,47], mothers and fathers in our sample had higher middle school completion rates (96% and 95%, respectively) than have previously been documented among parents in rural areas of China (66% and 53%, respectively) [75]. These characteristics would suggest the home language environment households in peri-urban areas may have higher rates of linguistic input and interaction in the home language environment than in rural areas, as the literature generally posits a positive relationship between SES and parental investments in the home language environment [3,5,6,14–18].

Like other studies[51,52], however, our descriptive analysis found lower counts of adult words, adult-child conversations, and lower child vocabularies compared to children in urban China [21,098 for AWC and 751 for CTC; 35]; they are also slightly lower than those in rural areas [14,739 for AWC and 611 for CTC; 36,37,39]. This seemingly contradicts the hypothesis that higher SES among peri-urban households relative to rural households would lead to stronger measures of the home language environment. It also suggests that peri-urban caregivers may face different challenges to effective parental investment than rural caregivers. Feng et al. (2023) identified socioeconomic and demographic predictors of stronger home language environment measures, including maternal employment and higher levels of household assets [51]. These associations suggest that higher SES indeed supports stronger home language environments. However, the authors suggest that these indicators may reflect higher levels of education or different attitudes towards communication, rather than a direct effect of economic status on investments [51].

Our follow-up qualitative interviews with 31 caregivers from the quantitative sample aimed to understand, in caregivers’ own words, how they invested in the home language environment and how economic constraints, time constraints, and knowledge constraints shaped those investments. The results found that the primary factor influencing parental investment in the home language environment is inaccurate parenting knowledge among caregivers. Although caregivers reported that they believed that parental interactions were important, many caregivers shared that they did not understand how to verbally engage with their children, and a large share of caregivers had misconceptions about how to effectively promote child language skill. These findings are consistent with past studies in rural areas of China, which have identified insufficient knowledge about interactive parenting as an underlying cause of cognitive delays among rural infants and toddlers [28,29,66,122,123]. For example, 14 of 31 caregivers expressed that physical growth was a more important aspect of child development during toddlerhood than language development. This may also explain why rates of adult works and adult-child conversation among peri-urban households have been found to increase with child age [51].

Our qualitative interviews also revealed that caregivers in peri-urban areas lacked reliable information sources on parenting and early language development, with most caregivers relying on information from friends, family, or social media rather than doctors, early childhood centers, or other credible sources. Although this phenomenon has also been documented in multiple studies in rural areas of China (Luo, Jia (74); Li, Rose (66), it is surprising that access to accurate information is limited in an urban setting, where parenting resources may be more easily available than in rural areas. This may be due to inequalities between migrant and local households in access to public early child education services [124,125]. Xu (125) found that due to China’s household registration system (which ties social services to one’s familial hometown), the number of free parenting services available to migrant caregivers was significantly lower than that of local parents. Without access to professionals, caregivers turn to nonprofessional sources like family members, friends, and other parents in the community, as well as the Internet [126,127].

In addition to a lack of credible information sources, we also found generational differences in parenting knowledge. Grandparent caregivers demonstrated less accurate knowledge than parents, and grandparents were more inclined to rely on their own intuition and experience to guide their parenting practices. This is consistent with previous research in rural areas of China, which has found grandmothers tend to engage in fewer interactive parenting practices than mothers [75]. Whereas only about a quarter of rural households have grandparent primary caregivers (26%; [75], grandparents were the primary caregivers for 43% of children in our quantitative sample. These findings point to a need for parenting resources to be made available to peri-urban households, particularly those in which grandparents have taken on caregiving responsibilities.

Time constraints presented as a less salient factor of investment for many households interviewed. About half of caregivers reported constraints on their time due to out-of-home employment, household chores, and caring for multiple children. Time constraints are not unique to caregivers in rural China, and the time constraints of primary caregiver employment somewhat contradicts the finding by Feng et al. (2023) that higher maternal employment was associated with greater counts of adult works and adult-child conversation [51]. One possibility is that time constraints compound the consequences of knowledge constraints for caregiver-child interaction. This is particularly relevant in cases where the primary caregiver (typically the mother) faces time contraints due to work outside the come, while the secondary caregiver (typically the grandmother) has limited knowledge of the importance of practice of language-stimulating interactions with toddlers. An alternative possibility, however, is that mothers who work may to place higher priority on the home language environment, leading to both higher counts of adult works and adult-child interaction and higher concern among caregivers about time constraints on interaction. This would suggest that maternal employment is a proxy for other characteristics, such as education or exposure to different parenting attitudes, that influence both parental investments and caregivers’ thinking about those investments.

In contrast, economic constraints did not emerge as a salient constraint among the households interviewed. This contradicts Feng et al. (2023)’s finding that higher levels of family assets were associated with higher adult work and conversational turn counts. This again suggests that there may be some variable confounding the relationship between family SES and the home language environment [51]. Considering the role of knowledge constraints and maternal education discussed above, this finding further suggests that caregiver education, and exposure to parenting knowledge through education or later employment, may support caregivers to provide robust home language environments.

The results of this study offer insights for supporting peri-urban caregivers to successfully invest in the language development of their young children. A key insight is the need for more credible sources of parenting information and support in peri-urban communities. One way to increase access to credible parenting information is through primary care providers: hospitals and clinics in peri-urban communities can provide additional information on the importance of interactive parenting and specific interactive parenting techniques, as well as dispel common misconceptions about child development. Another way to improve parenting knowledge is through community-based parenting interventions. A number of recent studies have evaluated curriculum-based parenting interventions in rural areas of China [28,123,128,129], including a community-based intervention that delivered parenting lessons in centrally located village parenting centers [128]. These interventions have been found to significantly improve the cognitive development of young children, yet their effects on language development are negligible, and no such intervention has yet been implemented in peri-urban areas.

In our study area, the research team has worked with local community institutions to establish a and maintain a community parenting center, and all households with children under 3 years (including those who participated in our study) were invited to attend. Many caregivers, especially younger mothers, expressed interest in receiving structured parenting support and training in the parenting center. Grandparent caregivers showed less interest in the parenting center or receiving parenting resources, however, reflecting a generational divide in parenting attitudes that we noted in our qualitative interviews. Additionally, when asked what types of parenting resources may be helpful, many caregivers were unable to answer. For these caregivers, a general community space dedicated to supporting their parenting may be more helpful than specific training or resources. Future research should investigate ways that these community-based interventions can better target improvements in the early home language environment, and whether these interventions can be effectively implemented in peri-urban settings.

## Conclusion

This study contributes to ECD research in China in two main ways. To date, the literature on the home language environment has focused mainly on Western settings or high-SES households in non-Western settings. This study helps to fill the gap in the literature on the home language environment by providing evidence from peri-urban households, a lower-SES, primarily rural group in urban China. Moreover, our study uses a rich quantitative data on the home language environment generated through the LENA recordings, which allows us to compare the home language environment of young peri-urban children compares to those in rural and urban areas. Second, our qualitative findings also contribute to the literature on parenting in China by identifying the factors of parental investments among peri-urban households and how they differ from rural areas. The results of this study can assist policy makers and practitioners to better support peri-urban caregivers of young children and maximize children’s development.

We also acknowledge limitations of this study. First, the quantitative and qualitative data were gathered from two peri-urban communities in southwest China. Although our final analytical sample of 81 households is relatively large compared to other studies using LENA (see, e.g., Zhang et al., 2015), the small sample size does have implications for the reliability and generalizability of the findings. Therefore, although our results offer preliminary evidence on the conditions of child language development in an under-studied setting, our results should be taken with caution, and the findings may not be generalizable beyond the study setting. Similarly, our qualitative interviews with 31 of the sample households should not be considered representative of peri-urban households.

Second, and more importantly, there are notable limitations used in the measures of the home language environment and child language skills in this study. Although the LENA system has been formally validated in Mandarin Chinese, a growing body of literature that has raised concerns about LENA’s validity [117,130,131]. Best practice continues to dictate that researchers supplement LENA-generated measures with manual annotations [132]. Unfortunately, due to limited resources on the part of the study team, manual annotation is not possible for this study. In addition to the LENA generated measures, the CDI measure assesses only children’s expressive language and not their expressive lagnuae, which may be more appropriate for this age range. We did not use the receptive language assessment in the CDI as this masure has only been validated for children age 8–18 months. There also may be bias in the CDI measure, as the measure relies on caregiver self-reporting of children’s vocabulary. Although the research team took measures to limit bias, this assessment may not be as accurate as a professional developmental assessment such as the Bayley Scales of Infant Development. Finally, the research team chose to focus specifically on adult caregiver investments in the home language environment, which neglects the role of child-child interactions in the language environment.

Finally, our qualitative analysis of the factors of parental investment focused on three factors that may constrain investments in the home language environment (economic constraint, time constraints, knowledge constraints). We did not examine other potential factors such as the role of child age, gender, prematurity or siblings which have been found to significantly predict differences in parental investments and early child development in other studies in China [51,133,134].

Despite these limitations, our study offers insight into the ways that caregivers in an under-studied and potentially vulnerable community invest in their children, and the obstacles they face to that investment in their daily lives. Future research should aim to rigorously assess all aspects of the home language environment and child language skills in peri-urban communities across China using gold standard assessment practices. Researchers should also continue to use mixed methods to understand both the characteristics of households that may predict the home language environment, and the mechanisms facilitating or constraining effective parental investments. Additional research is also needed to understand regional differences in parental investments and the home language environments of peri-urban households. Finally, future studies should develop and evaluate community interventions to support robust home environments and healthy early development for young peri-urban children.

## Supporting information

S1 Table Independent-sample t-test of LENA consent and LENA reject sub-samples. (DOCX)

S2 Table Independent-sample t-test of full quantitative sample qualitative subsample. (DOCX)

S3 Table Correlations between AWC, CTC and CDI. (DOCX)
