# Supplementary material for: Factors of parental investment in the home language environment in peri-urban China: A mixed methods study
Source: PLoS One. 2023 Nov 13;18(11):e0294158. doi: 10.1371/journal.pone.0294158 (PMC10642838; doi:10.1371/journal.pone.0294158)
Supplement: S1 Table — (DOCX) [file pone.0294158.s001.docx]

**Supporting Information**

S1 Table. Independent-sample t-test of LENA Consent and LENA Reject sub-samples

|  | LENA Consent | LENA Reject | Difference | |
| --- | --- | --- | --- | --- |
|  | (1) | (2) | (3) = (2) - (1) | |
| Child age (months) | 21.103 | 21.304 | 0.20 |  |
|  | [1.60] | [1.42] | (0.34) |  |
| Male child | 0.568 | 0.464 | -0.10 |  |
|  | [0.50] | [0.51] | (0.11) |  |
| Number of siblings | 0.123 | 0.179 | 0.06 |  |
|  | [0.33] | [0.39] | (0.08) |  |
| Mother is primary caregiver | 0.568 | 0.643 | 0.07 |  |
|  | [0.50] | [0.49] | (0.11) |  |
| Primary caregiver age (years) | 39.136 | 37.222 | -1.91 |  |
|  | [12.85] | [11.89] | (2.80) |  |
| Primary caregiver has ≥9 years of education | 0.519 | 0.643 | 0.12 |  |
|  | [0.50] | [0.49] | (0.11) |  |
| Primary caregiver reads books to the child | 0.593 | 0.714 | 0.12 |  |
|  | [0.49] | [0.46] | (0.11) |  |
| Primary caregiver sings to the child | 0.654 | 0.750 | 0.10 |  |
|  | [0.48] | [0.44] | (0.10) |  |
| Primary caregiver tells stories to the child | 0.519 | 0.500 | -0.02 |  |
|  | [0.50] | [0.51] | (0.11) | |
| Observations | 81 | 28 | 109 | |
| ^a^ Standard errors in parentheses, standard deviation in square brackets. | | | | |
| ^b^ *** p<0.01, ** p<0.05, * p<0.1 | | | | |
