# Supplementary material for: Factors of parental investment in the home language environment in peri-urban China: A mixed methods study
Source: PLoS One. 2023 Nov 13;18(11):e0294158. doi: 10.1371/journal.pone.0294158 (PMC10642838; doi:10.1371/journal.pone.0294158)
Supplement: S2 Table — (DOCX) [file pone.0294158.s002.docx]

**Supporting Information**

S2 Table. Independent-sample t-test of full quantitative sample qualitative subsample

|  | Quantitative  Sample  (n=81) | Qualitative  Sub-sample  (n=31) | Difference |
| --- | --- | --- | --- |
|  | (1) | (2) | (3) = (1) - (2) |
| Child age (months) | 21.10 | 21.19 | -0.09 |
|  | [1.60] | [1.58] | (0.34) |
| Male child | 0.57 | 0.58 | -0.01 |
|  | [0.50] | [0.50] | (0.11) |
| Mother is primary caregiver | 0.57 | 0.58 | -0.01 |
|  | [0.50] | [0.50] | (0.11) |
| Maternal age (years) | 29.11 | 28.87 | 0.24 |
|  | [4.69] | [4.89] | (1.00) |
| Mother has ≥9 years of education | 0.77 | 0.71 | 0.06 |
|  | [0.43] | [0.46] | (0.09) |
| Number of siblings in household | 0.12 | 0.16 | -0.04 |
|  | [0.33] | [0.37] | (0.07) |
| Household asset index score | 0.00 | -0.12 | 0.12 |
|  | [1.28] | [1.80] | (0.30) |
| Observations | 81 | 31 |  |
| ^a^ Standard error in parentheses, standard deviation in square brackets. | | | |
| ^b^ *** p<0.01, ** p<0.05, * p<0.1; no stars indicate no significant results. | | | |
