# Supplementary material for: Factors of parental investment in the home language environment in peri-urban China: A mixed methods study
Source: PLoS One. 2023 Nov 13;18(11):e0294158. doi: 10.1371/journal.pone.0294158 (PMC10642838; doi:10.1371/journal.pone.0294158)
Supplement: S3 Table — (DOCX) [file pone.0294158.s003.docx]

**Supporting Information**

S3 Table. Correlations between AWC, CTC and CDI

|  | Child vocabulary  (CDI z-score) | Child vocabulary  (CDI z-score) |
| --- | --- | --- |
|  | (1) | (2) |
| In_average AWC | 0.74*** |  |
|  | (0.28-1.21) |  |
| In_average CTC |  | 0.92*** |
|  |  | (0.54-1.30) |
| Constant | -6.93*** | -5.57*** |
|  | (-11.24 - -2.63) | (-7.89 - -3.24) |
| Observations | 81 | 81 |
| R-squared | 0.12 | 0.22 |
| ^a^ CI in parentheses | | |
| ^b^ *** p<0.01, ** p<0.05, * p<0.1 | | |
